# Causal Connectivity between the Human Anterior Intraparietal Area and Premotor Cortex during Grasp

**DOI:** 10.1016/j.cub.2009.11.063

**Published:** 2010-01-26

**Authors:** Marco Davare, John C. Rothwell, Roger N. Lemon

**Affiliations:** 1 Sobell Department of Motor Neuroscience and Movement Disorders, Institute of Neurology, University College London, Queen Square, London WC1N 3BG, UK; 2 Institute of Neuroscience, Laboratory of Neurophysiology, Université catholique de Louvain, B-1200 Brussels, Belgium

**Keywords:** SYSNEURO

## Abstract

The cortical visuomotor grasping circuit, comprising the anterior intraparietal area (AIP), ventral premotor (PMv), and primary motor cortex (M1) allows transformation of an object's physical properties into a suitable motor command for grasp [1–9]. However, little is known about how AIP contributes to the processing of grasp-related information conveyed through the cortical grasping circuit. We addressed this by studying the consequences of AIP “virtual lesions” on physiological interactions between PMv and M1 at rest or during preparation to grasp objects with either a precision grip or a whole-hand grasp. We used a conditioning-test transcranial magnetic stimulation (TMS) paradigm to test how PMv-M1 interactions [10–12] were modified by disrupting AIP function with theta-burst TMS (cTBS) [13]. At rest, AIP virtual lesions did not modify PMv-M1 interactions. In contrast, the usual muscle-specific PMv-M1 interactions that appeared during grasp preparation were significantly reduced following AIP cTBS without directly modifying corticospinal excitability. Behaviorally, disruption of AIP was also associated with a relative loss of the grasp-specific pattern of digit muscle activity. These findings suggest that grasp-related and muscle-specific PMv-M1 interactions are driven by information about object properties provided by AIP.

## Highlights

► Interactions between PMv and M1 are grasp specific ► AIP virtual lesions reduce grasp-related PMv-M1 interactions ► Changes in PMv-M1 connectivity affect hand muscle selectivity during grasp

## Results and Discussion

Human sensorimotor control is distinguished by the exquisite ability to grasp and manipulate objects and tools. To do this, visual information about an object's physical properties (e.g., size, shape, weight, slipperiness) must be transformed and used to select a motor command appropriate for efficient grasp. The key cortical circuit involved in this transformation involves the anterior intraparietal area (AIP), ventral premotor (PMv), and primary motor cortex (M1) [1–5, 8]. Thus, AIP contains neurons that discharge in relation to specific object properties [7], whereas many grasp-related “canonical” neurons [2] are found in PMv [6, 9, 14]. In addition, temporary inactivation of either AIP or PMv interferes with grasp performance [15–20]. Experiments in monkey [6, 7, 14] appear to show that an object's properties are encoded as a gradient along the AIP-PMv-M1 axis, with the object being first represented in visual attributes and then in terms of an appropriate grasp. If so, this arrangement suggests that AIP is a key area that provides PMv with information about an object's grasp-related properties such as size and shape [1, 2]. However, direct evidence for a causal input from AIP to the canonical grasp representations in PMv is still lacking.

**Figure 1:**
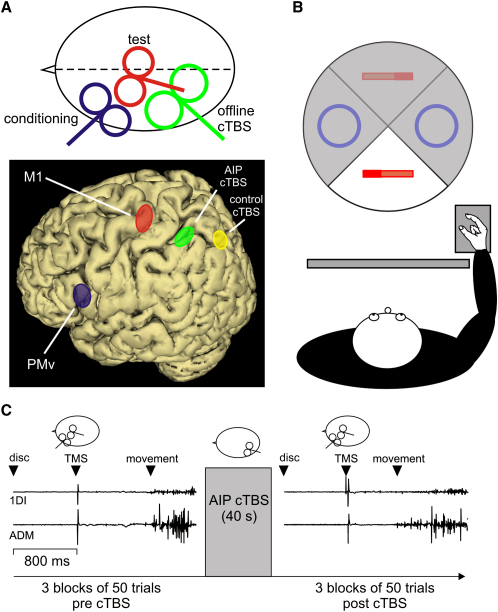
Trifocal Transcranial Magnetic Stimulation and Experimental Task (A) Coil orientations and location of the transcranial magnetic stimulation (TMS) sites as given by neuronavigation; the ventral premotor (PMv) (−56, 13, 18) is shown in blue, the primary motor cortex (M1) (−34, −25, 57) in red, the anterior intraparietal area (AIP) (−43, −39, 46) in green, and the control theta-burst TMS (cTBS) site in yellow. The ellipses show the 95% confidence interval centered over the mean calculated for all subjects (n = 9). See Supplemental Experimental Procedures for details. (B) Experimental task: subjects had to grasp objects at their own pace using either a precision grip between the index and thumb or a whole-hand grasp. A turntable randomly presented the objects 30 cm in front of the subject's hand pad. A screen, made from switchable transparent glass, was positioned between the subject and the turntable to allow precise timing of object presentation. (C) Experimental procedure: subjects performed 3 blocks of 50 trials before and after cTBS over AIP (or over the control area). Objects were presented in a random order, and TMS occurred 800 ms after object presentation (the disc, in this example). The TMS pulse was the go signal for subjects to start moving the hand, which occurred on average 700 ms later (see Supplemental Experimental Procedures). Typical recordings of the first dorsal interosseous (1DI) and abductor digiti minimi (ADM) are shown before and after cTBS over AIP.

The present experiments addressed this directly by testing whether AIP input is necessary for grasp-specific PMv-M1 interactions. We predicted that task-related interactions, but not those at rest, would be disrupted if we temporarily interfered with input from AIP by “offline” theta-burst transcranial magnetic stimulation (cTBS) (Figure 1A). Furthermore, if these task-related PMv-M1 interactions were functionally relevant, we expected to observe detectable consequences on grasp performance.

**Figure 2:**
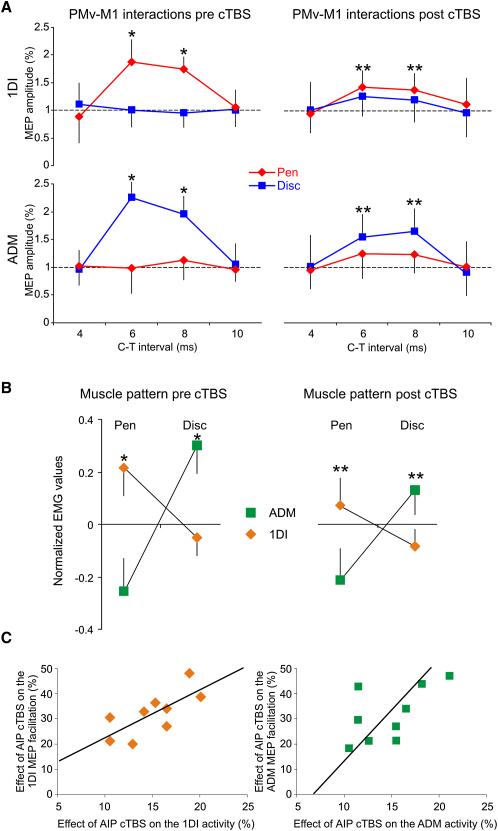
Effect of Anterior Intraparietal Area cTBS on PMv-M1 Interactions and on the Muscle Pattern (A) Relative amplitude of motor-evoked potentials (MEPs) (± standard deviation [SD]) recorded from the 1DI and ADM when preparing to grasp either the pen or the disc (left: before cTBS; right: after cTBS). y axis values represent the relative MEP amplitudes resulting from a suprathreshold test (T) stimulus applied over M1 preceded by a subthreshold conditioning (C) stimulus applied over PMv at different C-T intervals (x axis). Note that the facilitation of the 1DI when grasping the pen and of the ADM when grasping the disc (^∗^p < 0.05) decreased following cTBS (^∗∗^p < 0.05). (B) Z score normalized electromyographic (EMG) activity (±SD) measured during grasp of the pen or the disc (left: before cTBS; right: after cTBS). EMG activity was measured between the time at which subjects left the hand pad and 100 ms before the object liftoff. The 1DI was more active when grasping the pen compared to the disc and, conversely, the ADM was more active when grasping the disc compared to the pen (^∗^p < 0.05). Note the less-selective muscle pattern following AIP cTBS (^∗∗^p < 0.05). (C) Correlation between the effect of AIP cTBS on the MEP size and on the muscle pattern. The axes represent the effect of AIP cTBS in reducing the EMG activity (x axis) and the MEP facilitation (y axis) expressed in percent of values measured before the AIP cTBS. Left: 1DI values when grasping the pen; right: ADM values when grasping the disc. Note that the greater the disruptive effect of cTBS on MEP facilitation, the more the muscle pattern during grasp was disturbed.

Subjects were presented with two different objects, a pen or a disc (Figure 1B), which they had to lift with a precision grip or whole-hand grasp, respectively. The precision grip required more activity in the first dorsal interosseous (1DI) muscle than the whole-hand grasp; conversely, there was more abductor digiti minimi (ADM) muscle activity for the whole-hand grasp than the precision grip (repeated-measure analysis of variance [RM-ANOVA] grasp main effect: both F > 11.35, both p < 0.001; Figure 2B). This pattern was expected because 1DI is a prime mover in precision grip, whereas the ADM abducts the little finger to open the hand in the whole-hand grasp [21].

We first tested how PMv interacts with M1 by measuring the effect of a conditioning transcranial magnetic stimulation (TMS) pulse (C) applied over PMv on the muscle twitches (motor-evoked potentials [MEP]) produced by a test pulse (T) over M1 [10–12]. Stimuli were given after presentation of the object but before onset of movement (Figure 1C). With a C-T interval of 6–8 ms, MEPs produced in 1DI were facilitated when subjects prepared to grasp the pen (precision grip) but not when they prepared to grasp the disc (whole-hand grasp) (RM-ANOVA interval × grasp interaction: F = 7.32, p = 0.007; post hoc: both p < 0.004; Figure 2A). At the same C-T intervals, MEPs were larger in ADM when preparing the whole-hand grasp in comparison to preparing a precision grip (RM-ANOVA interval × grasp interaction: F = 6.78, p = 0.017; post hoc: both p < 0.015; Figure 2A). There were no significant effects at C-T intervals of 4 and 10 ms (all p > 0.05). The implication is that visual input about an object has been used to prepare a muscle-specific pattern of PMv-M1 interaction that is appropriate for the forthcoming grasp. PMv outputs would therefore exert facilitation on the M1 representation of selected muscles while leaving unaffected the corticospinal excitability of muscles not involved in the task (see also [12]).

Indeed, the difference in the MEPs recorded prior to the two grasps (MEP pen versus MEP disc, average of 6 and 8 ms C-T intervals) was correlated with the difference in electromyographic (EMG) activity during the subsequent grasp (EMG pen versus EMG disc; 1DI: r = 0.82, p = 0.003; ADM: r = 0.76, p = 0.001; see Figure S2A available online). This would be compatible with the idea that the pattern of PMv-M1 interactions might have a causal influence on the amount of activity in muscles used later to grasp the object.

cTBS-induced “virtual lesions” of AIP significantly depressed the grasp-specific differences in PMv-M1 interaction (RM-ANOVA main effect of cTBS: both F > 7.28, both p < 0.021). Thus, AIP lesions significantly reduced the PMv-M1 facilitation of 1DI seen during preparation of a precision grip (RM-ANOVA cTBS × interval × grasp interaction: F = 5.96, p = 0.032; Figure 2A), as well as the PMv-M1 facilitation of ADM during preparation of a whole-hand grasp (RM-ANOVA cTBS × interval × grasp interaction: F = 7.36, p = 0.027; Figure 2A).

AIP cTBS had no direct effect on corticospinal excitability, as measured by the amplitude of MEPs in response to pulses given alone over M1 (T pulses; RM-ANOVA cTBS main effect: F = 1.18, p = 0.23). Neither was there any effect on the PMv-M1 (inhibitory) interactions in subjects at rest (experiment 2; see Supplemental Experimental Procedures). Importantly, delivering cTBS over the medial segment of the intraparietal sulcus (control site) did not alter PMv-M1 interactions (all F < 1; Figure S1A). Because grasp-specific PMv-M1 interactions were attenuated by disruption of AIP, we suggest that under normal circumstances, AIP is necessary to supply grasp-related information to PMv.

Behaviorally, AIP virtual lesions disrupted the muscle pattern found when actually grasping the objects. Thus, 1DI was less active when grasping the pen and ADM was less active when grasping the disc compared to before cTBS of AIP (RM-ANOVA main effect of cTBS for 1DI and ADM: F = 8.32, p = 0.004 and F = 6.64, p = 0.011, respectively; Figure 2B). There was again no effect when delivering cTBS over the control site (all F < 1; Figure S1B). Interestingly, there was a correlation between the magnitude of the effect of AIP cTBS in reducing the grasp-specific PMv-M1 facilitation and its effect on the associated muscle pattern (1DI: r = 0.74, p = 0.004; ADM: r = 0.84, p = 0.001; Figure 2C). This suggests that an altered level of PMv-M1 interaction, secondary to a lack of AIP outputs, led to a less accurate muscle command that failed to shape the hand optimally when grasping different objects.

Despite the reduced modulation of EMG activity for each grasp, there was still a correlation between the grasp-related differences in PMv-M1 interaction and the muscle pattern during grasp (1DI: r = 0.81, p = 0.006; ADM; r = 0.75, p = 0.002; Figure S2B). However, the slopes of the regression lines across subjects were higher following AIP cTBS compared to before cTBS (RM-ANOVA main effect of cTBS: F = 11.35, p = 0.029; Figures S2A and S2B). This might result from some form of compensatory activity that attempts to maintain a selective muscle command despite disruption in underlying patterns of brain activity. For example, the reduced PMv outputs could influence M1 with a higher gain; alternatively, unaffected outputs from the dorsal premotor cortex could participate in the selection of the appropriate muscle response [22, 23].

The present study used trifocal TMS to investigate the contribution of AIP to functional interactions between PMv and M1 during grasping movements. As reported previously [12], our results show that the excitability of muscle-specific PMv-M1 connections is modulated differently during preparation to perform a precision grip versus a whole-hand grasp. The former increased facilitation between PMv and the 1DI representation in M1, whereas the latter increased facilitation between PMv and the ADM representation. The novel finding of the present study was that these effects were reduced after a virtual lesion of AIP produced by cTBS. Furthermore, there were behavioral consequences resulting from the AIP virtual lesion, because we found significant changes in the pattern of muscle activity when subjects actually grasped the objects. During precision grip there was less 1DI activation and during whole-hand grasp there was less ADM activation. In a previous study, we showed that such EMG changes were associated with subtle differences in grasping behavior [18].

We conclude that input from AIP is necessary to modulate PMv-M1 functional connectivity in a way relevant to the object being grasped. Importantly, the AIP contribution to excitability of connections between PMv and M1 is context specific, as well as grasp specific, because PMv-M1 interactions tested at rest were unaffected by disrupting AIP function. Finally, the fact that there was a linear relationship between the AIP-induced changes in PMv-M1 interaction and the change in muscle activity during the grasp suggests that input from PMv has a causal influence on patterns of muscle activity used in grasping objects. We suggest that a neural population in AIP that is activated by the vision of a particular graspable object provides PMv with grasp-related information that allows neurons in PMv to be tuned to the upcoming grasp. Transfer of this information to M1 in a muscle-specific manner enables PMv to contribute to the motor command required to grasp the object.

The effects of AIP virtual lesions were not due to nonspecific effects of cTBS. Targeting a control area with cTBS, medial to AIP, did not produce similar effects. In addition, the reduced grasp-specific MEP facilitation observed following AIP virtual lesions could not be explained by a general suppression of M1 outputs, because the corticospinal excitability was unchanged after cTBS. Finally, disrupting AIP function altered PMv-M1 interactions only during a grasp context but not at rest, which again strongly argues against a nonspecific effect of cTBS.

One possible explanation of the present results is that the AIP virtual lesion reduces the amount of visual information about the object that is passed to PMv, and this in turn reduces the extent to which motor programs can be tuned to different graspable objects. In other words, virtual lesions of AIP may reduce the “motor vocabulary” resident in PMv [2], affecting the precise hand-object relationship and resulting in less accurate “motor prototypes” to shape the hand appropriately around an object. This view suggests that each population of AIP neurons processes particular visual-related object properties [2, 7], such as size and shape (pen versus disc). This information, encoded in an “object” reference frame, is subsequently multiplexed into a “grasp” reference frame within PMv. The representation, at the level of single PMv neurons, of different object-related properties originating from AIP and their transformation into a motor reference frame that determines specific patterns of muscle activation for object grasp would fit with existing notions of “canonical neurons” within PMv [2]. The results of the present experiments suggest that individual canonical neurons have to receive the correct pattern of information from AIP so that the final grasp is matched to the properties of the object. Exactly how this matching is achieved is not known. One possibility is that it is learned through a process that associates the intrinsic properties of the object with the grasps that are effective in interacting with them [2].

**Figure 3:**
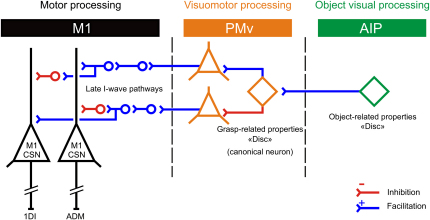
Schematic Model of Connectivity between AIP, PMv, and M1 PMv is connected with M1 corticospinal neurons (CSN; black pyramids) via indirect inhibitory (red) and facilitatory (blue) pathways (late I-wave pathways). The PMv output neurons (orange pyramids) giving rise to these pathways receive inhibitory and facilitatory connections from canonical neurons in PMv (orange diamond). Object-related neurons in AIP (green diamond) make facilitatory projections to canonical neurons in PMv. At rest, conditioning TMS over PMv reveals net inhibitory PMv-M1 interactions. When grasping the disc, the corresponding object-related neurons in AIP increase their firing rate, which facilitates in turn the appropriate canonical neurons in PMv. In this example, activation of the PMv canonical cell yields facilitation of the ADM muscle representation by inhibition of the PMv-M1 inhibitory connections and facilitation of the facilitatory PMv-M1 connections.

Based on the observed functional interactions between AIP, PMv, and M1 at rest and during grasping movements, we formulated a schematic model of the functional connectivity between these three areas that could explain our results (Figure 3). These connections are known to be relatively indirect and ultimately influence the later I waves of corticospinal activity generated within M1 [24, 25]. At rest, the canonical and object-related neurons in PMv and AIP have low firing rates; delivering a conditioning pulse over PMv reveals only a net inhibitory drive from PMv to M1 (Figure 3: red intracortical inhibitory inputs to M1 corticospinal neurons). AIP cTBS does not affect this net resting state inhibition because at-rest AIP neurons are mainly inactive. When subjects prepare to grasp an object (e.g., a disc), a population of AIP neurons that are tuned to those properties (i.e., size and shape of the disc [7, 14]) increase their firing rate. In the model, we assume that these AIP neurons provide information to selected neuronal populations in PMv (Figure 3, blue excitatory connections from AIP to PMv; see [2]). As a consequence of the facilitatory drive from AIP, this PMv “canonical” neuronal population increases its firing rate and provides both facilitatory PMv outputs to the M1 ADM representation and inhibitory drive to the 1DI representation. The overall balance between these two influences would result in a net facilitation of the appropriate muscle (ADM) representation (Figure 3, contrasting actions of PMv canonical neurons) and explains the grasp-specific facilitation of the M1 output representations (e.g., ADM when grasping the disc). Interestingly, a recent monkey study found that PMv-M1 connectivity could be either inhibitory or facilitatory [26]. In humans at rest, the predominant effect depends on the intensity of the conditioning stimulus delivered over PMv [27]. This indicates that different neural populations might be recruited by using different conditioning intensities and might explain how a net inhibitory drive at rest can switch to facilitation during movement preparation [11, 12], because the neural populations involved, and therefore their susceptibility to the conditioning PMv pulse, are different.

In the present study, because cTBS was applied offline over AIP, it still remains difficult to determine the directionality of the effects of AIP virtual lesions and hence the flow of information between AIP and PMv. A widely accepted view suggests that AIP provides PMv with grasp-related information [1, 2]. Alternatively, because of the reciprocal nature of the AIP-PMv connections [2, 28], it could be argued that canonical neurons in PMv have grasp-selective properties that depend upon recurrent feedback loops between PMv and AIP [1]. Because AIP contributes to online adjustments of grasp [29], recurrent loops between PMv and AIP could provide online control of the grasp-related information in PMv.

The present study is a specific example of the more general effects of disrupting function in one part of a complex system on activation in remote areas. It extends previous work in the field [30–33] by employing direct electrophysiological measures of functional connectivity between remote areas to test how these change after a virtual lesion of a third area. This is important because it shows that movement deficits following disruption of a cortical area “A” (for example) could result not from area A itself, but instead from an effect of that area on distant areas B or C or even on their respective interactions. Because a large frontoparietal network of areas is involved in the performance of hand movements [3–5, 8, 34], it could be that transient interference of one of these areas would yield interregional changes in connectivity within the cortical circuit [35].

In summary, the present results show that disruption of AIP impairs the normal changes in task-related interactions between PMv and M1 that prepare the hand muscles to grasp an object. This suggests that AIP is critical in processing context- and grasp-dependent information, which enables PMv to bias excitability levels in M1 hand representation during the preparation for an upcoming grasp. The triple-coil TMS approach used here allows us to investigate how one area contributes to the information flow through the cortical grasp network and potentially can be expanded in subsequent studies to provide high-resolution temporal information about the sequence of information transfer.

## Experimental Procedures

### Participants

Nine right-handed [36] subjects (20–33 years old) participated in the two experiments after providing informed consent and being screened for adverse reactions to TMS [37]. The experimental procedures were approved by the ethics committee of University College London.

### Transcranial Magnetic Stimulation

To investigate PMv-M1 interactions in the left hemisphere, we used two custom-made figure-eight coils (7 cm outer diameter) connected to two single-pulse monophasic Magstim stimulators. Neuronavigation was used to determine the sites of stimulation on individual anatomical magnetic resonance images previously gathered for each subject (Brainsight, Rogue Research; see Figure 1A; see also Supplemental Experimental Procedures). The C and T stimuli were set at 80% and 120% of the resting motor threshold, respectively (see Supplemental Experimental Procedures for details). AIP virtual lesions were produced by continuous theta-burst TMS for 40 s (cTBS: 3 pulses at 50 Hz every 200 ms; [13]) delivered via a 9 cm outer diameter coil connected to a rapid Magstim. cTBS was delivered “offline” after the session investigating control PMv-M1 interactions (Figure 1C). Then, 5 min after cTBS, we retested PMv-M1 with a second set of C-T stimuli. This delay period was chosen because a maximal inhibitory effect of cTBS over M1 is found after 5 min [13]. In addition, in experiment 1 only, to control for any nonspecific effects of cTBS over AIP, we targeted a more medial region of the intraparietal sulcus, where we delivered cTBS in a session with the same subjects around 1 week later (9 ± 2 days, mean ± standard deviation, n = 9), in which we again tested PMv-M1 interactions before and after control cTBS. In experiment 2, the same stimulation parameters were used.

### Experimental Design

In experiment 1, subjects had to perform 6 blocks of 50 trials, 3 before and 3 after the AIP (or control) cTBS (Figure 1C). The C-T intervals were 4, 6, 8, or 10 ms [12]. T alone was delivered in 1 out of 5 trials, and the MEP amplitudes measured in this condition were used as baseline values. The pen and the disc were presented randomly with a 0.5 probability. In experiment 2, subjects were at rest. Two blocks of 50 trials were acquired before and after the AIP cTBS. The C-T intervals were as described for experiment 1.

### Data Acquisition and Analysis

The peak-to-peak amplitude of each individual MEP was measured and expressed as a percentage of the control (baseline) MEP (T stimulus alone) gathered during the same block. Trials in which any EMG activity was present during the movement preparation period (800 ms) were discarded. The muscle activity involved in the preshaping of the hand during either precision grip or whole-hand grasp was estimated by computing the area-under-curve of the rectified EMG between the time at which subjects left the hand pad and 100 ms before the object liftoff. For each muscle and each subject, the EMG values were Z score normalized to the grand average of each subject (both grasps). See Supplemental Experimental Procedures for more details.

### Statistical Analyses

In experiment 1, for each site of cTBS application (AIP or control), RM-ANOVAs were performed on the reaction and movement times, relative MEP amplitudes and EMG values with C-T interval (4, 6, 8, 10, or T alone), grasp (precision grip or whole-hand grasp), and cTBS (pre or post) as within-subject factors. In experiment 2, RM-ANOVAs were performed on the relative MEP size with C-T interval (4, 6, 8, 10, or T alone) and cTBS (pre or post) as within-subject factors. Planned post hoc comparisons (each C-T interval with respect to T alone) were performed via Dunnett's test. Correlations between the amount of MEP facilitation and EMG activity were performed via the Pearson procedure.

## Supplemental Information

Document S1. Supplemental Experimental Procedures and Two Figures
