# Supplementary material for: Causal Connectivity between the Human Anterior Intraparietal Area and Premotor Cortex during Grasp
Source: Curr Biol. 2010 Jan 26;20(2):176–81. doi: 10.1016/j.cub.2009.11.063 (PMC2824111; doi:10.1016/j.cub.2009.11.063)
Supplement: Document S1. Supplemental Experimental Procedures and Two Figures [file mmc1.pdf]

Current Biology, Volume *20*

Supplemental Information

Causal Connectivity between the Human Anterior Intraparietal Area and Premotor Cortex during Grasp

Marco Davare, John C Rothwell, and Roger N Lemon

Supplemental Results

AIP cTBS did not affect reaction (RT) or movement (MT) times (ANOVA main effect of cTBS, INTERVAL or GRASP: all $F < 1$). Across conditions, the RT and MT were 734.5 ± 107.9 and 1242.5 ± 227.8 ms, respectively (mean ± SD, n = 9). This indicates that altering PMv-M1 connectivity by AIP cTBS did not affect the reaching phase of movements.

When subjects were at rest (experiment 2), we found that AIP virtual lesions did not change the resting state connectivity between PMv and M1 (ANOVA, main effect of cTBS: $F = 1.16$, $p = 0.21$). Delivering a conditioning pulse over PMv led to smaller MEPs in the 1DI and ADM, but only for C-T intervals of 6 or 8 ms (ANOVA, main effect of INTERVAL: both $F > 7.32$, both $p < 0.002$). This data confirms our previous study [1] and further indicates that AIP has no influence, at rest, on the excitability of short latency interactions between PMv and M1.

Experimental Procedures

Experimental Task

In experiment 1, subjects were asked to grasp objects at their own pace using either a precision grip (PG) between the index and thumb or a whole hand grasp (WHG). The two objects were a 2 cm diameter pen grasped with a PG and an 11 cm diameter disc grasped with a WHG. The two objects had the same mass (50 g). The objects were presented in a random order by means of a motorised turntable connected to a CED 1401 (Cambridge Electonic Design, Cambridge, UK) (see Figure 1B and [2] for details). The duration of visible presentation of the object was controlled by a screen (switchable transparent glass, All Brilliant Tech, Beijing) placed between the subject and the turntable. Subjects could see the object to grasp only after the screen was made transparent. A TMS pulse (see below), occurring 800 ms after object presentation [3], was the cue to release the hand-pad and grasp the object appropriately. Subjects had then to lift it to approximately 5 cm height and replace it on the turntable after a beep. When subjects repositioned the right hand on the hand-pad, the screen turned opaque and the next trial started

after a randomised inter-trial time (7-10 s). In experiment 2, subjects were at rest comfortably seated in an armchair.

Transcranial Magnetic Stimulation

To investigate PMv-M1 interactions in the left hemisphere, we used two custom-made figure-of-eight coils (7 cm outer diameter) connected to two single-pulse monophasic Magstim model 200 stimulators (Magstim Company, Whitland, UK). The conditioning (C) stimulus was delivered over PMv, with anterior to posterior induced current, through a coil held tangentially to the skull with the handle pointing forward; the test (T) stimulus was delivered over M1, with posterior to anterior induced current, through a coil held perpendicularly to the central sulcus with the handle pointing backwards (Figure 1A). The C and T stimuli were set, respectively, at 80% and 120% of the resting motor threshold (rMT) [4], defined as the minimum intensity that induced motor evoked potentials (MEPs) ≥ 50 μV peak-to-peak in both the first dorsal interosseous (1DI) and abductor digiti minimi (ADM) in 5 out of 10 trials [5]. The rMT was determined by using a coil connected to a single-pulse Magstim stimulator and equalled on average 42±6 % of the maximal stimulator output (mean ± SD, n = 9).

To perform AIP "virtual lesions" on the same (i.e. left) hemisphere, we used a continuous theta-burst TMS for 40 s (cTBS: 3 pulses at 50 Hz every 200 ms; [6]). We connected a 9 cm outer diameter coil to a rapid Magstim (Magstim Company, Whitland, UK). cTBS was delivered "offline" after the session investigating control PMv-M1 interactions. Then, 5 min after cTBS had been applied, we retested PMv-M1 with a second set of C-T stimuli. This delay period was chosen since Huang et al. (2005) found a maximal inhibitory effect of cTBS over M1 after 5 min. In addition, in experiment 1 only, to control for any unspecific effects of cTBS over AIP, we targeted a more medial region of the intraparietal sulcus where we delivered cTBS in a session in the same subjects around one week later (9 ± 2 days, mean ± SD, n = 9) in which we again tested PMv-M1 interactions before and after control cTBS. In experiment 2, the same stimulation parameters were used.

Stimulation Sites

The coil position was precisely determined, in every subject, by means of a neuronavigation technique of the stimulation sites onto individual anatomical magnetic resonance images previously gathered for each subject (Brainsight, Rogue, Montreal). In order to target PMv, the coil was positioned over the caudal portion of the pars opercularis of the inferior frontal gyrus. In the present study, the mean normalized MNI coordinates of PMv were -56 ± 2, 13 ± 6, 18 ± 7 mm (x, y, z, mean ± SD; n = 9), close to those reported by functional imaging studies [7-9]. Additionally, we have shown that a "virtual lesion" of this region impairs precision grasping [10] and is functionally connected to M1 [1].

In order to target M1, the coil was positioned over the site where TMS induced the largest MEPs in both 1DI and ADM. The coregistration procedure confirmed that the M1 site overlapped the hand knob [11]; its mean normalized MNI coordinates were -34 ± 4, -25 ± 3, 57 ± 11 mm (x, y, z, mean ± SD; n = 9), which are also comparable to those reported in functional imaging studies [12, 13]. The mean Euclidian distance between PMv and M1 stimulation sites was 60 ± 9 mm

(mean ± SD; n = 9), a distance sufficient to allow positioning of both coils over the same hemisphere (Figure 1A).

To target AIP, the coil was positioned at the intersection between the intraparietal sulcus and postcentral sulcus. A recent fMRI study has shown that visually guided grasping movements selectively and invariably activated, in every subject, an area located at the junction of these two sulci [14]. The mean normalized MNI coordinates of the left AIP stimulation sites were, -43 ± 7, -39 ± 7 and 46 ± 4 mm (x, y, z; mean ± SD; n = 9). These coordinates overlapped the location of AIP reported by fMRI studies in humans [7, 8, 14, 15]. Finally, the control cTBS site was located on a more medial part of the intraparietal sulcus, on average 46 ± 7 mm (mean ± SD; n = 9) medially to AIP.

Data Acquisition and Analysis

The Magstim stimulators were triggered using Spike2 software and the CED data acquisition interface (Cambridge Electronic Design, Cambridge, UK). EMG activity was recorded with bipolar surface electrodes (belly-tendon), one pair positioned over the 1DI and the other over ADM. The raw EMG signals were amplified (1K; Neurolog, Digitimer Ltd, UK) and digitized at 5 kHz for offline analysis.

The reaction time (RT) was defined as the delay between the TMS pulse (C stimulus) and the hand-pad release. The movement time (MT) was computed as the delay between the hand-pad release and object lift-off, detected by means of an optical sensor placed just below the turntable. The peak-to-peak amplitude of each individual MEP was measured and expressed as a percentage of the control (baseline) MEP (T stimulus alone) gathered during the same block. Trials where any EMG activity was present during the movement preparation period (800 ms) were discarded. The muscle activity involved in the preshaping of the hand during either the PG or WHG was estimated by computing the area-under-curve of the rectified EMG between the time at which subjects left the hand-pad and 100 ms before the object lift off. For each muscle and each subject, the EMG values were Z-score normalized to the grand average of each subject (both grasps).

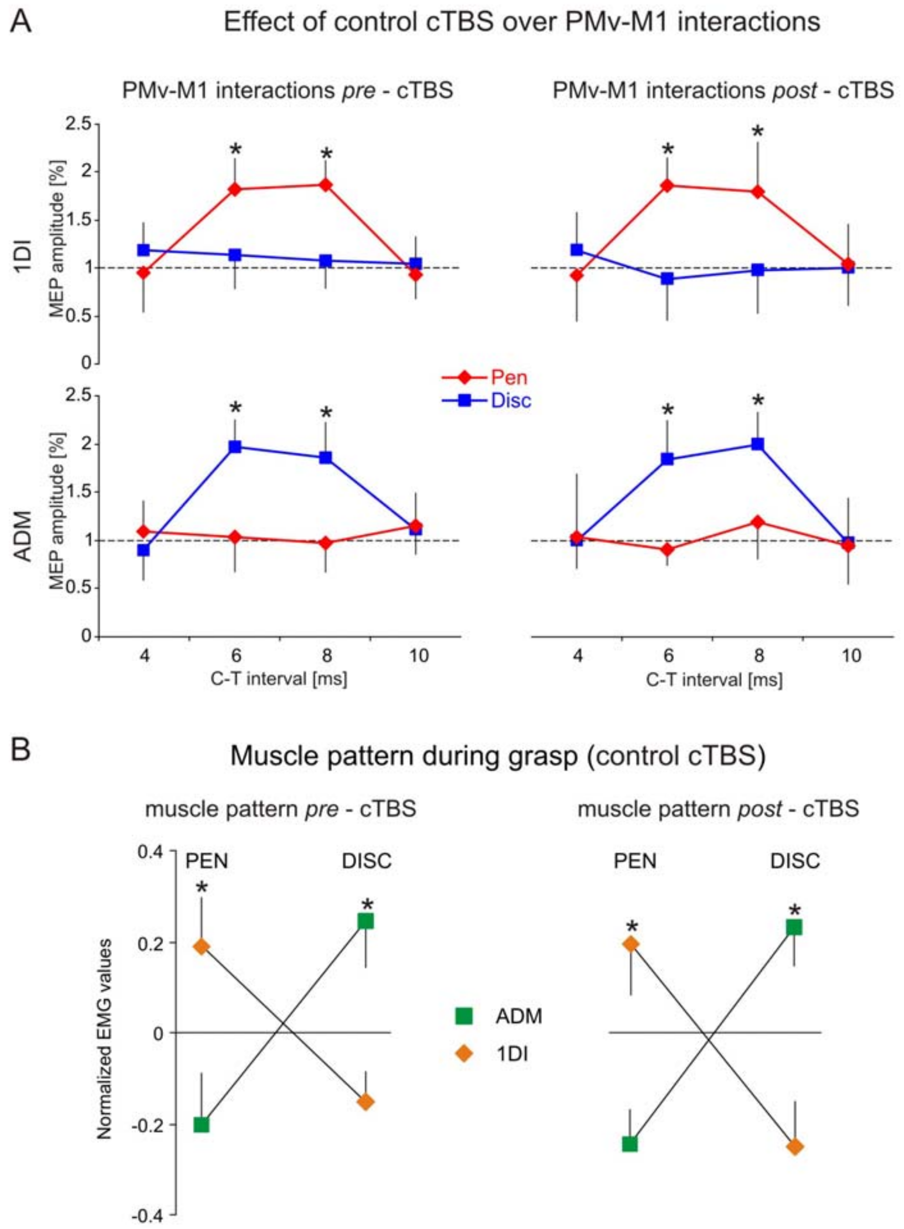

Figure S1. Effect of Control cTBS on PMv-M1 Interactions and the Muscle Pattern
(A) Relative amplitude of MEPs (±SD) recorded from the 1DI and ADM while subjects were preparing to grasp either the pen or the disc. Values on the y axis represent the relative MEP amplitudes resulting from a supra-threshold test (T) stimulus applied over M1 preceded by a sub-threshold conditioning (C) stimulus applied over PMv at different C-T intervals (x axis). Note that following cTBS of the control site there was no reduction in the facilitation of the 1DI when grasping the pen, and of the ADM when grasping the disc (*$p < 0.05$).
(B) Z score normalized EMG activity (±SD) measured during grasp of the pen or the disc. EMG activity was measured between the time at which subjects left the hand-pad and 100 ms before the object lift-off. The 1DI was more active when grasping the pen compared to the disc and conversely for the ADM (*$p < 0.05$). Note that cTBS over the control site does not change the muscle pattern.

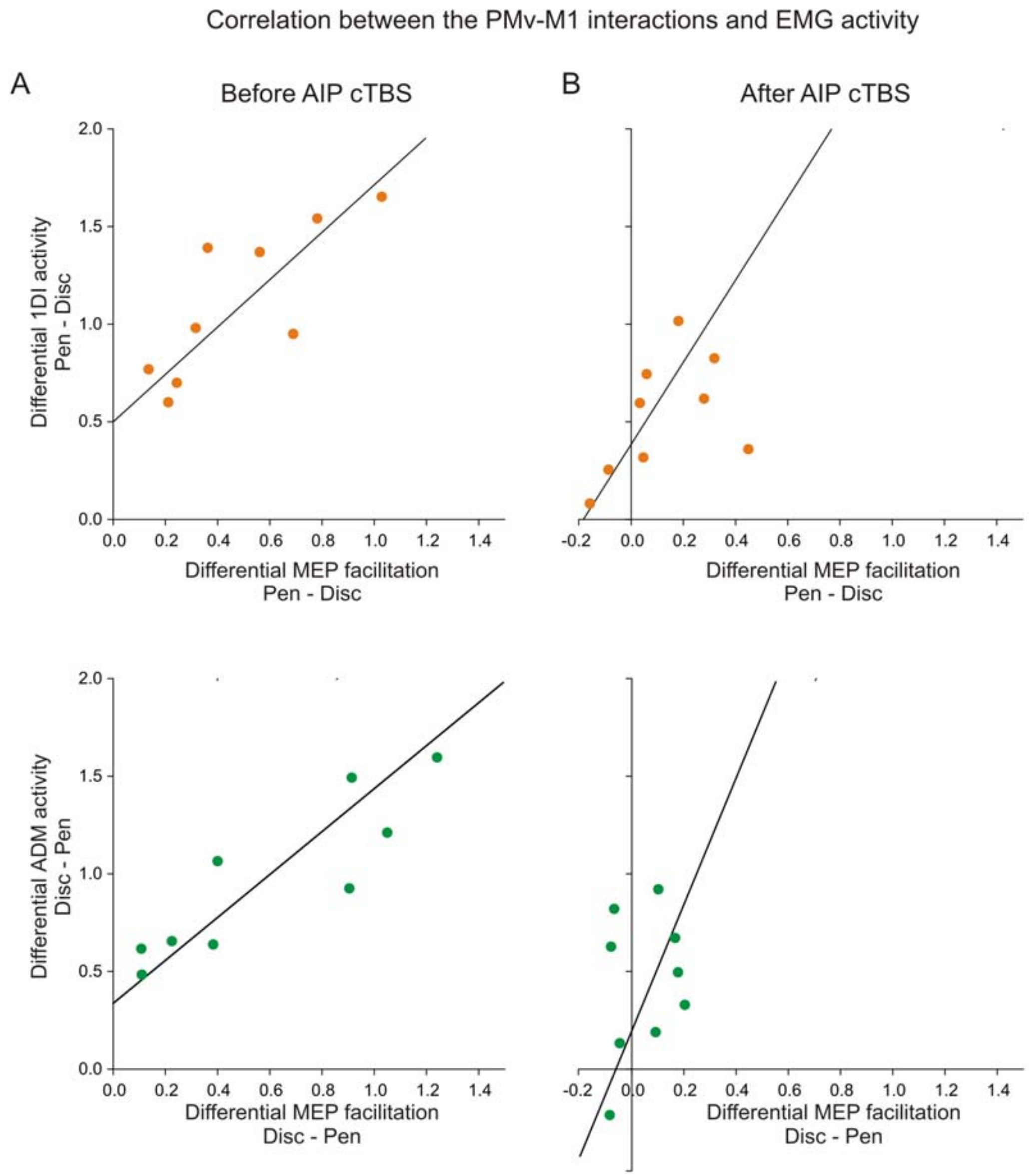

Figure S2. Effect of AIP cTBS on the Amount of MEP Facilitation Related to the Muscle Pattern

Correlations between the differential MEP facilitation and the muscle activity during grasp (from hand-pad release to 100 ms before object lift-off). Values of MEP facilitation gathered from C-T intervals of 6 and 8 ms (mean amount of facilitation in each subject at these two C-T intervals). The x axis shows the difference between the MEP amplitude recorded while subjects (n = 9) prepared to grasp either the disc or the pen (A for 1DI: MEP pen – MEP disc; B for ADM: MEP disc - MEP pen). The y axis represents the difference in EMG activity between grasps of the two objects (EMG pen – EMG disc for 1DI or EMG disc - EMG pen for ADM). Note that the slope of the regression line is steeper following AIP cTBS.

References

1. Davare, M., Lemon, R., and Olivier, E. (2008). Selective modulation of interactions between ventral premotor cortex and primary motor cortex during precision grasping in humans. J Physiol *586*, 2735-2742.
2. Davare, M., Montague, K., Olivier, E., Rothwell, J.C., and Lemon, R.N. (2009). Ventral premotor to primary motor cortical interactions during object-driven grasp in humans. Cortex.
3. Prabhu, G., Voss, M., Brochier, T., Cattaneo, L., Haggard, P., and Lemon, R. (2007). Excitability of human motor cortex inputs prior to grasp. J Physiol.
4. Civardi, C., Cantello, R., Asselman, P., and Rothwell, J.C. (2001). Transcranial magnetic stimulation can be used to test connections to primary motor areas from frontal and medial cortex in humans. Neuroimage *14*, 1444-1453.
5. Rossini, P.M., Barker, A.T., Berardelli, A., Caramia, M.D., Caruso, G., Cracco, R.Q., Dimitrijevic, M.R., Hallett, M., Katayama, Y., Lucking, C.H., et al. (1994). Non-invasive electrical and magnetic stimulation of the brain, spinal cord and roots: basic principles and procedures for routine clinical application. Report of an IFCN committee. Electroencephalogr Clin Neurophysiol *91*, 79-92.
6. Huang, Y.Z., Edwards, M.J., Rounis, E., Bhatia, K.P., and Rothwell, J.C. (2005). Theta burst stimulation of the human motor cortex. Neuron *45*, 201-206.
7. Binkofski, F., Buccino, G., Posse, S., Seitz, R.J., Rizzolatti, G., and Freund, H. (1999). A fronto-parietal circuit for object manipulation in man: evidence from an fMRI-study. Eur J Neurosci *11*, 3276-3286.
8. Ehrsson, H.H., Fagergren, E., and Forssberg, H. (2001). Differential fronto-parietal activation depending on force used in a precision grip task: an fMRI study. J Neurophysiol *85*, 2613-2623.
9. Kuhtz-Buschbeck, J.P., Ehrsson, H.H., and Forssberg, H. (2001). Human brain activity in the control of fine static precision grip forces: an fMRI study. Eur J Neurosci *14*, 382-390.
10. Davare, M., Andres, M., Cosnard, G., Thonnard, J.L., and Olivier, E. (2006). Dissociating the role of ventral and dorsal premotor cortex in precision grasping. J Neurosci *26*, 2260-2268.
11. Yousry, T.A., Schmid, U.D., Alkadhi, H., Schmidt, D., Peraud, A., Buettner, A., and Winkler, P. (1997). Localization of the motor hand area to a knob on the precentral gyrus. A new landmark. Brain *120 (Pt 1)*, 141-157.
12. Fink, G.R., Frackowiak, R.S., Pietrzyk, U., and Passingham, R.E. (1997). Multiple nonprimary motor areas in the human cortex. J Neurophysiol *77*, 2164-2174.
13. Picard, N., and Strick, P.L. (2001). Imaging the premotor areas. Curr Opin Neurobiol *11*, 663-672.
14. Frey, S.H., Vinton, D., Norlund, R., and Grafton, S.T. (2005). Cortical topography of human anterior intraparietal cortex active during visually guided grasping. Brain Res Cogn Brain Res *23*, 397-405.
15. Begliomini, C., Wall, M.B., Smith, A.T., and Castiello, U. (2007). Differential cortical activity for precision and whole-hand visually guided grasping in humans. Eur J Neurosci *25*, 1245-1252.
